# Cardiac motion and its dosimetric impact during radioablation for refractory ventricular tachycardia

**DOI:** 10.1002/acm2.13925

**Published:** 2023-02-06

**Authors:** Joseph Harms, Eduard Schreibmann, Neal S. Mccall, Michael S. Lloyd, Kristin A. Higgins, Richard Castillo

**Affiliations:** ^1^ Department of Radiation Oncology University of Alabama at Birmingham Birmingham Alabama USA; ^2^ Department of Radiation Oncology Winship Cancer Institute of Emory University Atlanta Georgia USA; ^3^ Section of Clinical Cardiac Electrophysiology Emory University Atlanta Georgia USA

**Keywords:** cardiac radioablation, deformable image registration, motion management, SBRT, ventricular tachycardia

## Abstract

**Introduction:**

Cardiac radioablation (CR) is a noninvasive treatment option for patients with refractory ventricular tachycardia (VT) during which high doses of radiation, typically 25 Gy, are delivered to myocardial scar. In this study, we investigate motion from cardiac cycle and evaluate the dosimetric impact in a cohort of patients treated with CR.

**Methods:**

This retrospective study included eight patients treated at our institution who had respiratory‐correlated and ECG‐gated 4DCT scans acquired within 2 weeks of CR. Deformable image registration was applied between maximum systole (SYS) and diastole (DIAS) CTs to assess cardiac motion. The average respiratory‐correlated CT (AVG_resp_) was deformably registered to the average cardiac (AVG_cardiac_), SYS, and DIAS CTs, and contours were propagated using the deformation vector fields (DVFs). Finally, the original treatment plan was recalculated on the deformed AVG_resp_ CT for dosimetric assessment.

**Results:**

Motion magnitudes were measured as the mean (SD) value over the DVFs within each structure. Displacement during the cardiac cycle for all chambers was 1.4 (0.9) mm medially/laterally (ML), 1.6 (1.0) mm anteriorly/posteriorly (AP), and 3.0 (2.8) mm superiorly/inferiorly (SI). Displacement for the 12 distinct clinical target volumes (CTVs) was 1.7 (1.5) mm ML, 2.4 (1.1) mm AP, and 2.1 (1.5) SI. Displacements between the AVG_resp_ and AVG_cardiac_ scans were 4.2 (2.0) mm SI and 5.8 (1.4) mm total. Dose recalculations showed that cardiac motion may impact dosimetry, with dose to 95% of the CTV dropping from 27.0 (1.3) Gy on the AVG_resp_ to 20.5 (7.1) Gy as estimated on the AVG_cardiac_.

**Conclusions:**

Cardiac CTV motion in this patient cohort is on average below 3 mm, location‐dependent, and when not accounted for in treatment planning may impact target coverage. Further study is needed to assess the impact of cardiac motion on clinical outcomes.

## 1 INTRODUCTION

Ventricular tachycardia (VT) is an arrhythmia which is commonly associated with functional and structural heart disease and is a leading cause of cardiac death.^1^ The majority of VTs present in patients with cardiac abnormalities due to reentry within myocardial scar tissue.^2^ With the modest efficacy of anti‐arrhythmic pharmacotherapy, the standard of care treatment for VT is radiofrequency (RF) catheter ablation (CA) of the VT substrate. However, the efficacy of CA is inversely correlated with the severity of heart disease and the number of prior RF attempts, with reported success rates ranging from 55% to 89%.^3^ For patients with medically refractory VT who fail CA, there are few therapeutic options. More recently, cardiac radioablation (CR), which delivers a highly conformal dose of ionizing radiation to a myocardial scar volume, has emerged as an effective option, decreasing VT burden by 69%–99% in multiple case series and a single phase I/II clinical trial.^4^, ^5^, ^6^, ^7^, ^8^, ^9^, ^10^

Early success has resulted in a growing number of medical centers implementing CR.^11^ While a uniform dose prescription regimen is used, there is variability in pre‐treatment imaging, target delineation, and motion management techniques.^12^ Additionally, while there is vast experience with respiratory motion management in thoracic radiation oncology, there are remaining unknowns about the interplay between cardiac and pulmonary motion. Preliminary motion studies in both healthy patients and those with arrhythmias have used either MRI,^13^, ^14^ ECG‐gated CT,^15^ or respiratory‐gated CT.^16^ Most of these studies have focused on healthy patient populations and have analyzed cardiac cycle substructure movement. In a study of 10 patients receiving ECG‐gated CT in the context of transcatheter aortic valve replacement, Ouyang et al found that more than 90% of measured displacements among substructures during the cardiac cycle were less than 5 mm.^15^ In a study of 18 patients undergoing magnetic resonance‐guided radiotherapy for intrathoracic tumors, Morris et al found directional displacements of the cardiac chambers greater than 5 mm in up to 20% of patients, with median displacements around 2 mm as seen on cardiac MRI even under voluntary breath hold.^13^

There are limited studies into both cardiac substructure and target volume motion, along with assessment of their potential impact on radiation dosimetry in patients with refractory VT. A better and thorough understanding of cardiac and cardiopulmonary motion management will be needed for larger, multi‐institutional trials and widespread CR adoption. A recent study by Prusator et al used rigid registration to investigate target motion for CR treatments using both ECG‐gated CT and respiratory‐gated CT.^17^ For nine of the 11 patients included in their study, the ECG‐gated CT revealed greater target motion in at least 1 translational direction than in the respiratory‐gated CT. In this study, we present motion analysis from eight patients treated with CR for refractory VT. To account for volume changes and deformation expected during the cardiac cycle, deformable image registration was applied to both ECG‐gated CT and respiratory‐averaged 4DCT to investigate voxel‐wise cardiac motion and the impact of this motion on target coverage.

## 2 METHODS

Patients reported in this study were all treated under the compassionate use mechanism under the direction and approval of the Institutional Review Board at Emory University. Between April 2018 and December 2020, 21 patients were treated with CR for refractory VT. All patients received respiratory‐correlated 4DCT as part of standard‐of‐care radiation therapy planning for thoracic stereotactic body radiation therapy (SBRT). Eight patients also had contrast‐enhanced ECG‐gated CT acquired by radiology within 2 weeks of their CT simulation, with phase data at maximum diastole (DIAS) and maximum systole (SYS) as well as 4D CTs averaged over the cardiac cycle (AVG_cardiac_) included in their medical records. The cardiac scans were acquired with the patient in head‐first‐supine position and under voluntary breath hold. Radiation therapy simulation included acquisition of contrast‐enhanced 3D free‐breathing CT (FBCT) of the thorax in addition to non‐contrast respiratory‐gated 4DCT. All patients also received at least one electrophysiology study with electroanatomic mapping. Target delineation was then performed on the 3D FBCT with the consensus of the treating electrophysiologists, a cardiac imaging specialist, and the treating radiation oncologist.^7^

Twelve distinct myocardial scars, which we will call clinical target volumes (CTVs) in keeping with radiation oncology nomenclature, were included in this dataset. Two patients had two targets and another had three targets. Of these 12 volumes, nine were epicardial, wrapping around the left ventricle, and three were endocardial, in various locations within the heart. Margins expansions of 0–5 mm were used to generate planning target volumes (PTVs). Anatomical characteristics and volumetric information for all targets are provided in Table 1. While a respiratory 4D internal target volume (ITV) approach is typically used in curative treatment of lung tumors, this was not the approach used for patients treated with CR. Instead, the targets were drawn with some manual margin, as determined by consensus among the treatment team. This manual margin accounted for uncertainty in both the electrophysiological map reading and the spatial registration of the EP map to the planning CT. Then, the final CTV to PTV margins were made on a patient‐by‐patient basis. Generally, smaller targets, and patients with multiple targets received larger target expansions. Patient 1 for example had three targets, all below 5 cm^3^ and in distinct anatomic locations, so 5 mm margins were used for this target. Patient 3 had large CTVs, but since there were multiple CTVs treated with a single isocenter, a 3 mm margin was applied. Patient 8 had the largest CTV, aside from patient 3, and no margin was used for this treatment.

**TABLE 1:** Target volume characteristics for patients included in analysis.

PATIENT	TARGET LOCATION	PTV MARGIN (MM)	CTV VOLUME (CM^3^)	PTV VOLUME (CM^3^)
1A	Epicardial	5	3.0	13.4
1B	Epicardial	5	4.1	18.3
1C	Endocardial	5	1.0	8.9
1^a^		5	8.1	40.5
2	Epicardial	1	51.6	86.6
3A	Epicardial	3	12.4	28.8
3B	Endocardial	3	81.9	140.4
3^a^		3	94.3	169.3
4A	Endocardial	2	2.9	9.8
4B	Epicardial	2	8.5	26.5
4^a^		2	11.4	36.3
5	Epicardial	3	12.9	28.4
6	Epicardial	1	15.1	24.4
7	Epicardial	2	47.0	95.3
8	Epicardial	0	69.1	69.1

*Note*: For patients with multiple target volumes, these are denoted by letters.

^a^ Combined target volumes.

The target and cardiac chamber structures were propagated from the free‐breathing CT to the averaged 4D respiratory CTs (AVG_resp_) for treatment planning and dose calculation. Other thoracic OARs, such as esophagus and lungs were delineated on the AVG_resp_. All patients were treated with 25 Gy in one fraction, with 95% of the PTV receiving prescription dose and maximum doses between 27.5 and 35 Gy. Treatment planning was performed in Eclipse (Varian Medical Systems, Palo Alto, CA) with dose calculated by the Anisotropic Analytic Algorithm (AAA) version 15.6. All treatments were planned with volumetric modulated arc therapy (VMAT) using 6 MV flattening filter free beams. Seven patients were treated with two partial arcs spanning 180–210 degrees, and patient three was treated with two separate isocenters and two partial arcs per isocenter because of separation between the CTVs. The slice thickness for the AVG_resp_ CT images was 2mm for all cases, and the dose calculation grid size was 2 mm.

**FIGURE 1:**
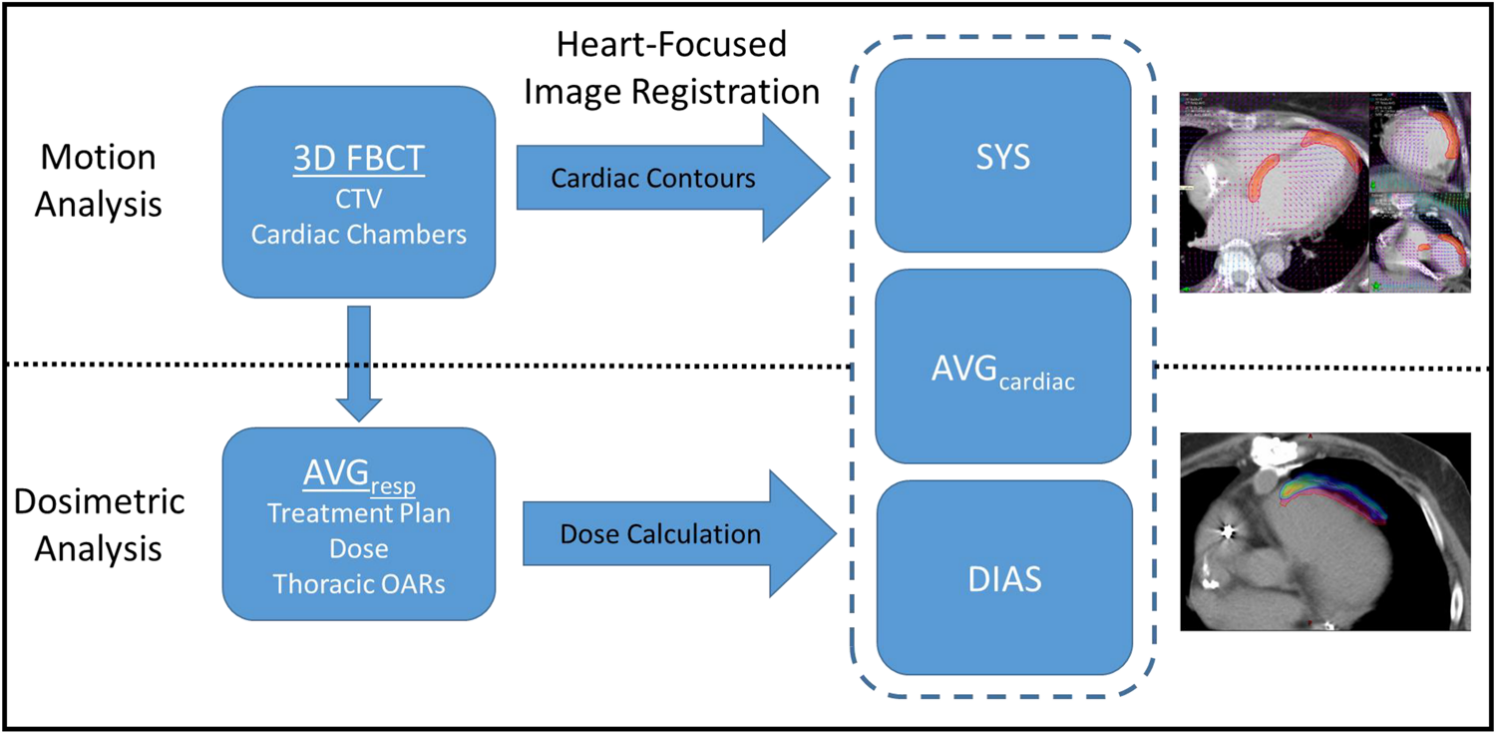
Description of the image registration workflow used in this study. For treatment planning, the CTV and cardiac contours were delineated on the 3D free‐breathing CT (FBCT). These then followed a cardiac‐focused image registration process for propagation of cardiac contours to the systolic (SYS), diastolic (DIAS), and average cardiac (AVG_cardiac_) 4DCT datasets, respectively, for motion analysis. For dosimetric analysis, the average respiratory 4D CT (AVG_resp_) was deformably registered to each of the cardiac CT datasets and dose was recalculated on this deformed CT. The images in the right column are representative of each of the cardiac CT datasets.

For this study, all radiation therapy data, including the non‐contrast respiratory‐gated 4DCT and contrast‐enhanced 3D FBCT images, structure sets, and treatment fields were imported into Velocity (Varian Medical Systems, Palo Alto, CA), along with the available contrast‐enhanced ECG‐gated CTs. A series of image registrations was then applied as outlined in Figure 1, first for motion assessment and subsequently for dosimetric comparison. For cardiac motion analysis, the FBCT was rigidly registered to the AVG_cardiac_ in a multi‐step process, first focusing on bony anatomy. Once the bony anatomy was as close as possible, the rigid registration was refined by focusing on a volume of interest around the heart. Any shifts present in this initial rigid registration were assumed to be due to setup differences and thus ignored in subsequent analysis. Finally, the FBCT was deformably registered to the AVG_cardiac_ CT, with a volume of interest focusing on the heart and including margin of a few centimeters in the lungs and chestwall. This allowed the DIR to be driven by differences in cardiac position rather than global differences. Structure contours (CTV, left and right atria, left and right ventricles) were then propagated to the cardiac CTs according to the deformation vector field (DVF). This registration process was repeated separately for both the SYS and DIAS image sets. Independently, to investigate cardiac cycle motion alone, the DIAS dataset was deformably registered to the SYS dataset and DVFs were exported along with structure sets to be analyzed in Matlab (Mathworks, Natick, MA). The DVFs contain deformations in Cartesian coordinates for each CT voxel. Since the two images are at the extremes of the cardiac cycle, the DVF can be used to analyze cardiac cycle motion magnitude and direction, as well as to create motion‐volume histograms. Structure contours were converted to binary masks and DVFs lying within these masks were used for structure‐specific analysis. Means and standard deviations of the separate DVFs were used to estimate motion magnitude in the superior/inferior (SI), medial/lateral (ML), and anterior/posterior (AP) directions. Total motion is calculated as the vector sum of the directional motion. All registrations were carried out in Velocity using the deformable multi‐pass option. This is a B‐spline registration algorithm that is applied first at a coarse voxel level. This coarse registration is then used as initial condition for a subsequent registration at a finer level, and this process is repeated one more time for three total passes.^18^

After cardiac motion assessment, a similar DIR workflow was applied from the AVG_resp_ to the AVG_cardiac_ for dosimetric assessment. Deforming the AVG_resp_ onto the AVG_cardiac_ allows for creation of a deformed planning CT (DPCT) where the Hounsfield units (HU) are propagated from the AVG_resp_ dataset to the AVG_cardiac_ dataset, allowing for dose calculation using HU from the treatment planning CT (AVG_resp_) and the anatomy as reflected in the AVG_cardiac_ CT. The original treatment plan is then recalculated on the DPCT with fixed Monitor Units. This process was then repeated using the DIAS and SYS CTs as the target images.

The use of the DVF to propagate contours has been previously implemented for adaptive dosimetric calculations.^19^, ^20^ For further validation of this method for motion analysis in this application, a radiation oncologist retrospectively delineated both atria and ventricles on the DIAS and SYS datasets for all eight patients. Similarity metrics, including Dice similarity coefficient (DSC), centroid mean distance (CMD), and mean surface distance (MSD) were calculated between propagated and manually segmented contours. Due to the expertise and clinical context required to delineate CTVs, these were not re‐contoured on the cardiac datasets and the DIR‐propagated contours alone were used for analysis. For this analysis of DIR measures, the manual contours were treated as ground truth. The goal of using DIR in this work was to measure target motion over the cardiac cycle. As there is no ground truth for CTV motion, direct quantification of the DIR uncertainty in the CTV is not possible. Instead, the motion measured via CMD for the manual contours was treated as a reference surrogate, and the discrepancy between this measurement and the motion measured by evaluating the mean of the DVF between DIR‐propagated contours can be used for uncertainty quantification.

## 3 RESULTS

To validate our method of DVF contour propagation, the DVF‐propagated contours and manually generated contours were compared for similarity. These two sets of cardiac chamber contours generally showed high levels of overlap, with (average ± SD) DSCs of 0.86 ± 0.05 across all images and an average volume difference of 1.5% (2.15 cm^3^) ± 13.3% (30.4 cm^3^), as shown in Table 2. Table 3 shows the distance between the centroid of the manual contours and DVF‐generated contours. Over all directions, the CMD was 1.7 ± 1.3 mm, with similar differences in each direction. The smallest discrepancy was between the left ventricles, most likely because largest emphasis for qualitative review of the registrations was placed on the LV and surrounding myocardium. Table 4 shows the difference between the ground truth chamber motion, as measured by the CMD between manual contours delineated in max systole and max diastole, and the chamber motion measured by evaluating the mean of the DVF between the DIR‐propagated contours. Mean agreement in any direction was within 1 mm and standard deviations within 3 mm. The average difference in measured shift over all chambers and all directions was 0.5 mm. While the DVF‐generated contours tend to be less smooth than manual contours and can have some discontinuities around structure borders, this analysis shows that DVF‐generated contours proved to be an adequate surrogate for manual contours in this setting.

**TABLE 2:** Quantitative contour comparisons between the DVF‐generated contours and manually delineated contours.

	LA	LV	RA	RV	AVERAGE
DSC	0.85 ± 0.04	0.83 ± 0.04	0.91 ± 0.01	0.84 ± 0.04	0.86 ± 0.05
VD (%)	11.4 ± 10.5	−4.4 ± 7.6	4.1 ± 15.4	−5.0 ± 11.9	1.5 ± 13.3
VD (CM^3^)	21.8 ± 19.5	−15.7 ± 33.9	9.5 ± 29.3	−7.0 ± 24.6	2.2 ± 30.4
MSD (MM)	4.4 ± 4.3	4.2 ± 4.0	3.1 ± 2.5	3.7 ± 3.4	3.9 ± 3.6

Abbreviations: DSC, dice score coefficient; MSD, mean surface distance; VD, volume difference.

**TABLE 3:** Centroid mean distance (CMD) between DVF‐generated contours and manually delineated contours.

	LA	LV	RA	RV	AVERAGE
ML	1.4 ± 0.9	1.5 ± 0.9	2.3 ± 1.3	1.1 ± 0.9	1.6 ± 1.1
AP	1.9 ± 1.5	1.6 ± 1.3	1.6 ± 0.8	2.4 ± 1.8	1.9 ± 1.3
SI	2.2 ± 2.0	1.2 ± 0.6	1.5 ± 0.9	2.2 ± 2.1	1.8 ± 1.5
TOTAL	1.8 ± 1.5	1.4 ± 0.9	1.8 ± 1.0	1.9 ± 1.7	1.7 ± 1.3

**TABLE 4:** Mean difference in mm in motion measurements using the CMD between manual contours and the mean value of the DVF for DIR‐generated contours.

	LA	LV	RA	RV	AVERAGE
ML	−0.2 ± 0.8	1.0 ± 1.7	2.1 ± 3.6	−1.3 ± 1.3	0.7 ± 2.5
AP	−0.3 ± 1.2	0.2 ± 1.6	−0.5 ± 1.5	2.7 ± 4.2	0.8 ± 2.9
SI	−0.5 ± 1.2	−0.2 ± 1.0	0.2 ± 0.9	0.1 ± 0.8	0.0 ± 1.0
TOTAL	−0.3 ± 1.1	0.4 ± 1.4	0.9 ± 2.7	0.9 ± 3.2	0.5 ± 2.3

The mean motion magnitude during the cardiac cycle for each of the cardiac chambers and the target volumes are shown in Table 5. The CMD for each structure is shown along with the mean and standard deviation of the DVFs. The cardiac chambers all showed largest motion in the superior/inferior (SI) direction, with mean values between 1.9 and 3.1 mm, while motion in the medial/lateral (ML) and anterior/posterior (AP) directions were generally ⩽2 mm as measured with the DVFs. CTV motion ranged between 0.0 and 5.2 mm in any cardinal direction, with average total magnitudes less than 5 mm. Only one patient (patient 1) exhibited target motion in any one direction above 5 mm, with 5.2 mm magnitude motion in the ML direction. Three target volumes (1C, 2, and 6) had total magnitudes above 5 mm. There was no correlation between CTV size and motion in any direction. Generally, motion measured with the DVF was larger than that measured by the CMD. This is to be expected as certain voxels can experience large deformations which may heavily weight the mean of the DVF without greatly moving the structure centroid.

**TABLE 5:** Cardiac motion magnitudes in mm for cardiac chambers and the CTV as measured by the mean of the DVF and the CMD.

		LA	LV	RA	RV	CTV
ML	DVF	1.1 (0.0‐1.9)	0.8 (0.3‐2.0)	1.4 (0.5‐3.7)	2.0 (1.2‐2.8)	1.7 (0.0‐5.2)
	CMD	1.8 (0.2‐3.8)	1.5 (0.2‐2.5)	2.2 (0.3‐4.6)	1.8 (0.2‐4.6)	0.5 (0.3‐0.8)
AP	DVF	1.3 (0.2‐2.3)	1.3 (0.4‐3.2)	2.2 (0.3‐3.4)	1.1 (0.3‐2.2)	2.4 (0.5‐3.9)
	CMD	1.3 (0.0‐2.6)	1.3 (0.3‐2.6)	1.3 (0.2‐2.5)	1.0 (0.2‐2.8)	1.9 (0.4‐3.2)
SI	DVF	3.1 (0.1‐11.0)	2.7 (0.6‐5.7)	2.4 (0.6‐8.7)	1.9 (0.3‐3.8)	2.1 (0.4‐4.6)
	CMD	2.1 (0.1‐5.3)	1.2 (0.1‐2.4)	1.6 (0.1‐2.9)	2.3 (0.3‐6.7)	1.9 (0.1‐4.9)
TOTAL	DVF	4.2 (1.7‐11.4)	4.4 (3.5‐6.3)	4.6 (2.3‐8.9)	4.2 (3.1‐5.0)	4.3 (1.8‐7.2)
	CMD	3.5 (0.3‐5.8)	2.6 (1.3‐6.8)	3.3 (1.6‐5.2)	3.5 (1.3‐6.8)	3.3 (2.4‐5.0)

*Note*: Values shown are mean (range) over all patients.

**FIGURE 2:**
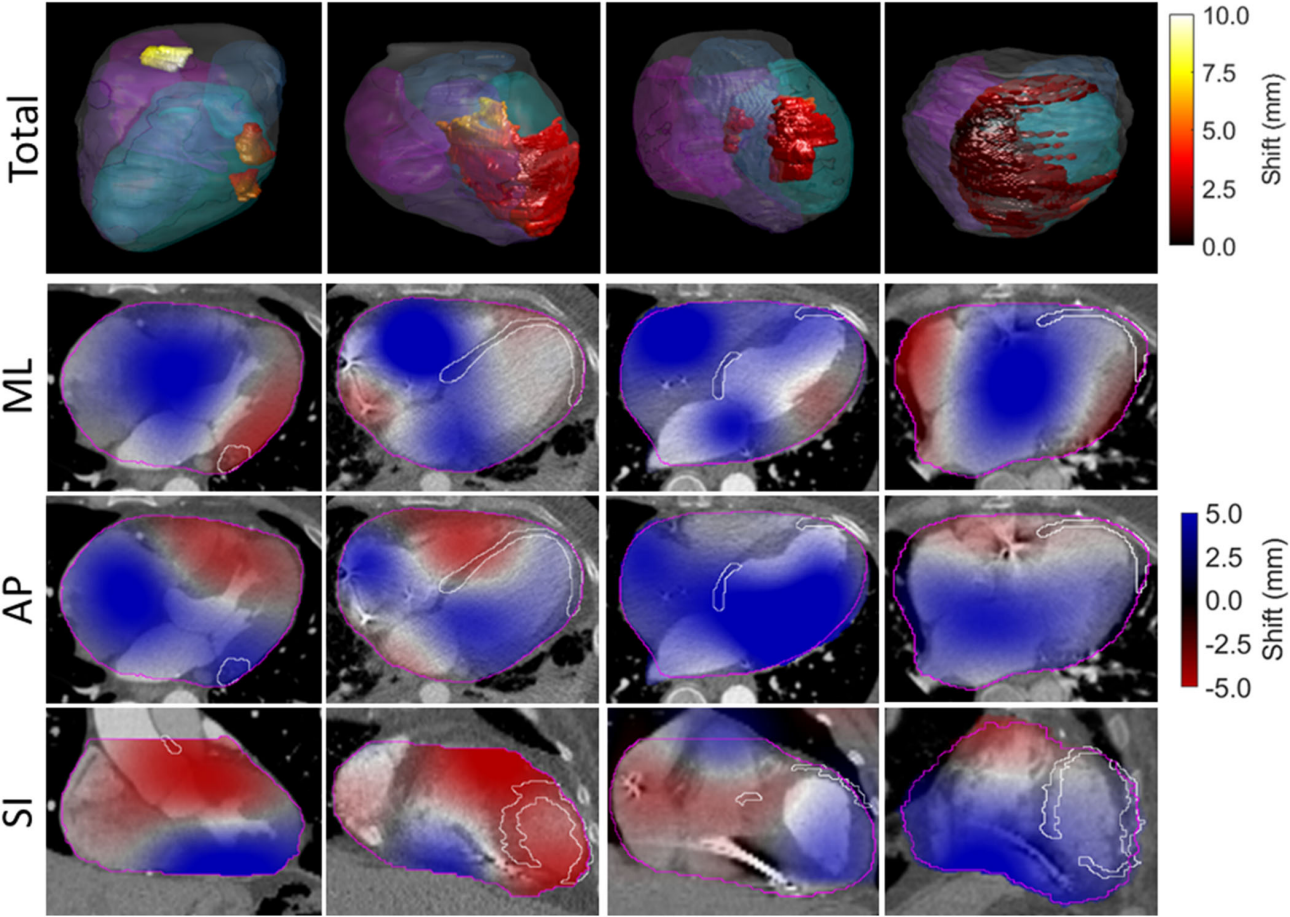
3D representation of target anatomy and motion magnitudes from four example patients. The top row shows the cardiac anatomy with the left and right atria and ventricles shaded in a blue/purple gradient (left ventricle is shown in cyan). CTV contours are overlaid with a heat‐map indicating the magnitude of cardiac motion. The second row shows the deformation vector field (DVF) in the medial/lateral (ML) direction with positive values indicating deformation to the patient's left, third row shows anterior/posterior (AP) DVFs with positive values indicating anterior deformation, and the final row shows superior/inferior (SI) DVFs with positive values indicating superior deformation.

Figure 2 shows a 3D representation of target anatomy, with heat maps overlaid to show the magnitude of cardiac motion within the CTV. The first column (patient 1) shows three distinct target volumes. The two inferior posterolateral CTVs show total motion magnitudes less than 5 mm, while the smaller superior target between the ascending aorta and pulmonary artery has an overall target motion near 10 mm. For the second patient with a much larger target volume (patient 7), the directional analysis shows that this U‐shaped target moves in different directions depending on location, with the endocardial portion moving anteriorly and medially and the epicardial region moving posteriorly during the cardiac cycle. Columns 3 and 4 show patients 4 and 8, respectively. Although patient 8 has the largest CTV of all the patients shown in this figure, motion across the entire target volume is small. Patients 7 and 8 were more representative of both target volumes and motion magnitudes seen in this cohort.

Table 6 shows the average difference in position of the CTV between the AVG_resp_ and AVG_cardiac_ scans. Effectively, this represents the difference in target position when cardiac motion is not incorporated into treatment planning. The average shifts per patient between the AVG_resp_ and AVG_cardiac_ are shown along with the maximum shift from AVG_resp_ to either the SYS or DIAS image, representing maximum displacement of the CTV during the cardiac cycle with respect to the planning CT. Between these image sets, AP and ML directional motion were similar, while SI motion dominated, as expected.

**TABLE 6:** Displacement between ECG‐ and respiratory‐gated CTs for the CTVs as measured in mm by the mean of the DVF.

	AVG_RESP_‐AVG_CARDIAC_	AVG_RESP_‐MAX_CARDIAC_
ML	2.0 ± 1.0	3.0 ± 1.0
AP	1.8 ± 1.3	2.3 ± 1.5
SI	4.2 ± 2.0	4.3 ± 2.6
TOTAL	5.8 ± 1.4	7.6 ± 2.6

*Note*: Values are presented as means ± standard deviations over all patients in the cohort.

**FIGURE 3:**
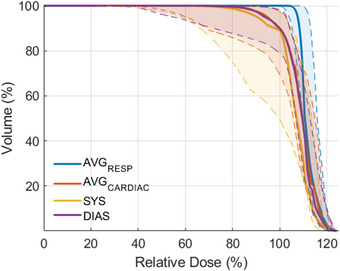
Median DVHs from taken from all CTVs. The shaded region for each DVH corresponds to the interquartile range.

**FIGURE 4:**
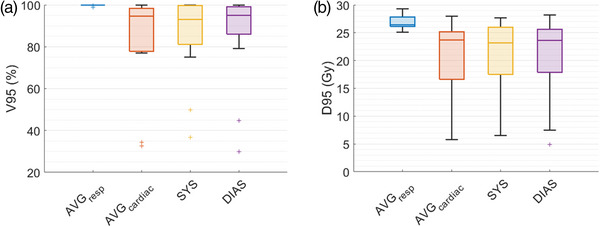
Boxplots showing the volumes receiving 95% coverage (a) and doses to 95% of the target volumes (b) for all individual CTVs.

**FIGURE 5:**
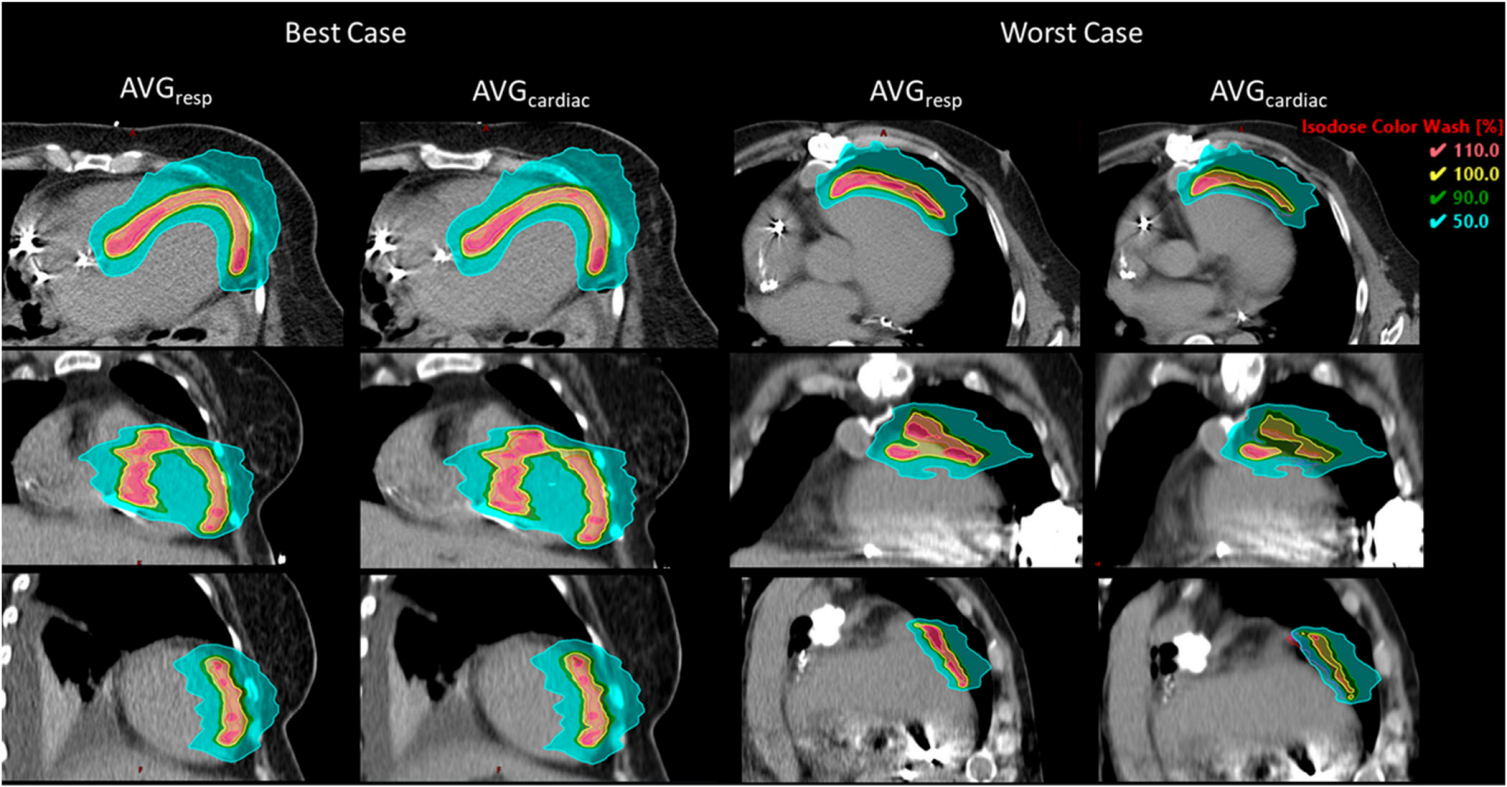
3D dose distributions calculated on the AVG_resp_ and AVG_cardiac_ images for the best and worst cases in terms of CTV V95 on the AVG_cardiac_ dataset. All slices are shown near the center of the target, with axial slices in the top row, coronal slices in the middle row, and sagittal slices in the final row.

Finally, we analyzed the potential dosimetric impact of cardiac motion. Figure 3 shows the median CTV DVH across all targets, with the shaded regions showing the interquartile range. Table 7 shows dose coverage for all patients for the nominal plans (AVG_resp_) and the plans recalculated on the DPCT as deformed to the AVG_cardiac_, SYS, and DIAS datasets, respectively, and these data are shown visually in the boxplots in Figure 4. For five of the patients, decreases in V95 and D95 were small, with an average decrease in coverage of 3.6% and a maximum of 10.2%. For patients 1, 6, and 8, dose to the target volume was decreased, with 95% of the volumes receiving an average dose of 13.5 Gy. This leads to the drop in the DVH shoulder for the cardiac imaging sets seen in Figure 3. Figure 5 shows the best‐ and worst‐ case scenarios in terms of dose coverage on the AVG_cardiac_ dataset. In the worst‐case scenario (patient 6), shifts of 1.6 mm ML, 3.9 mm AP, and 2.5 mm SI led to the CTV moving partially out of the high dose region. DVH metrics specified by the RAVENTA trial protocol^21^ for the heart and lungs are reported in Table 8 along with mean and max doses to each of the cardiac chambers. As the amount of motion was relatively small, there is little change in the dose to 50% of the heart or lungs. The average dose to the hottest 5% of the lungs tended to increase on the cardiac datasets as motion along the lung‐heart interface led to greater high‐dose spill in the lungs. No consistent differences were seen in max dose to any of the cardiac chambers.

**TABLE 7:** CTV dose coverage on the nominal plan (AVG_RESP_) and plans recalculated on ECG‐gated CTs.

	V95 (%)				D95 (GY)			
PATIENT	AVG_RESP_	AVG_CARDIAC_	SYS	DIAS	AVG_RESP_	AVG_CARDIAC_	SYS	DIAS
1A	100.0	100.0	98.4	100.0	28.8	28.0	25.4	28.2
1B	100.0	78.4	91.3	79.2	29.3	11.2	20.9	12.5
1C	100.0	32.6	49.8	29.9	28.0	5.8	6.5	4.9
1^a^	100.0	67.8	77.6	63.1	28.3	7.4	8.4	6.2
2	100.0	95.1	91.1	94.3	25.8	23.8	22.6	23.4
3A	100.0	94.6	100.0	94.0	27.7	23.7	27.7	23.3
3B	98.9	94.4	87.3	96.7	26.5	23.4	19.5	24.9
3^a^	99.1	94.5	88.9	96.7	26.5	23.4	20.0	24.9
4A	100.0	100.0	100.0	99.9	26.4	25.9	26.3	25.8
4B	100.0	94.8	99.3	95.7	26.2	23.7	25.6	23.9
4^a^	100.0	96.0	99.4	96.5	26.3	24.2	25.7	24.2
5	100.0	96.8	100.0	99.6	26.9	24.6	26.8	26.2
6	100.0	34.4	36.7	92.9	26.1	14.7	14.5	23.3
7	100.0	100.0	94.9	98.7	26.2	25.8	23.7	25.4
8	99.7	77.1	75.1	44.8	25.1	18.5	15.5	7.5
MEAN	99.8	80.7	82.5	81.2	27.0	20.5	19.6	19.3

*Note*: Only individual target volumes are included in the mean dose calculation for patients with multiple CTVs. Mean value is calculated per target and excludes combined target volumes.

^a^ Combined target volumes.

**TABLE 8:** Median and interquartile ranges for selected DVH metrics for the lungs, heart, and cardiac chambers.

		AVG_RESP_	AVG_CARDIAC_	SYS	DIAS
LUNGS	D1500cm^3^ (Gy)	0.2 (0.1‐0.4)	0.2 (0.1‐0.6)	0.2 (0.0‐0.7)	0.1 (0.1‐0.6)
	D50% (Gy)	0.5 (0.2‐0.7)	0.5 (0.2‐0.7)	0.5 (0.3‐0.7)	0.5 (0.2‐0.7)
	D5% (Gy)	4.9 (2.9‐6.8)	5.3 (3.1‐6.7)	5.5 (3.1‐7.0)	5.5 (2.8‐6.9)
HEART	D50% (Gy)	1.8 (1.1‐4.7)	2.2 (1.1‐4.4)	1.8 (1.0‐4.4)	2.0 (1.1‐4.4)
LEFT ATRIUM	Mean (Gy)	2.8 (2.4‐4.5)	3.4 (2.6‐4.6)	2.8 (2.1‐4.4)	2.8 (2.5‐4.6)
	Max (Gy)	13.2 (9.2‐28.6)	16.2 (9.2‐25.3)	12.8 (6.6‐28.2)	11.4 (9.2‐29.0)
RIGHT ATRIUM	Mean (Gy)	2.1 (1.1‐3.3)	2.1 (1.1‐3.4)	1.9 (1.1‐3.5)	2.2 (1.1‐3.3)
	Max (Gy)	6.6 (4.3‐17.1)	6.7 (5.4‐15.6)	6.9 (5.2‐18.0)	6.9 (5.4‐14.8)
LEFT VENTRICLE	Mean (Gy)	8.2 (5.2‐9.7)	7.9 (5.5‐10.1)	7.3 (3.6‐10.2)	7.9 (5.1‐9.5)
	Max (Gy)	30.3 (29.4‐30.8)	31.1 (30.6‐31.3)	30.2 (29.0‐31.3)	30.5 (29.7‐31.4)
RIGHT VENTRICLE	Mean (Gy)	3.8 (3.0‐7.0)	3.5 (2.4‐7.3)	3.5 (2.2‐7.1)	3.4 (2.8‐6.8)
	Max (Gy)	29.4 (27.7‐30.1)	29.1 (27.0‐30.5)	29.5 (23.5‐30.1	29.8 (27.0‐30.6)

## 4 DISCUSSION

CR has emerged as a noninvasive, often effective treatment option for patients with refractory VT. As CR becomes more widely adopted across different treatment centers, a better understanding of cardiac motion is necessary. This study was a preliminary investigation into cardiac motion using ECG‐gated CT in a cohort of eight patients treated with CR. Motion magnitudes averaged over target volumes were often less than 5 mm. Motion was found to be non‐uniform and varied from patient to patient. In this study, based on cardiac motion alone, CTV coverage may have been impacted for three out of eight patients. Further study is needed to assess the potential clinical impact that cardiac motion may have on VT burden, if any.

The dose delivered for CR is 25 Gy in a single fraction and was originally postulated to create radiation‐induced fibrosis. However, clinical efficacy so far has been shown within 4–8 weeks,^5^, ^6^, ^7^, ^8^ a timeline faster than expected for fibrosis to explain the reduction in VT events. A recent study by Zhang et al suggests that increased Na_v_1.5 and connexin 43 in irradiated hearts allow for electrical conduction reprogramming.^22^ Notably, the current prescription dose of 25 Gy in a single fraction was determined from preclinical data to create radiation‐induced fibrosis,^23^ and Zhang et al found that 20 Gy may also be sufficient to achieve therapeutic benefits of RT for VT reduction. It is possible that this lower dose threshold would mean that small motion effects which move target volumes out of the 25 Gy isodose line, may have limited effect on clinical outcomes because targets still receive more than 20 Gy and this dose is sufficient for VT reduction.

There are multiple options for motion mitigation in radiation oncology. Deep inspiration breath hold, phase‐gated beam delivery, 4D‐delineation of ITVs, and abdominal compression are all common techniques, especially in thoracic SBRT.^24^ The data presented in this work suggest these existing techniques may be adequate for some CR patients, but not all patients, and the extent of cardiac motion needs to be understood prior to treatment. Ho et al described a strategy in which phase gating from 40% to 60% based on the respiratory signal is employed if respiratory displacement is greater than 6 mm or the target volume is within 2 cm of the stomach.^4^ This approach also relies on using the cardiac lead as an accurate fiducial for tracking target motion. Our study suggests that motion is often non‐uniform, and it is reasonable to surmise a cardiac lead may either over or under‐estimate movement. Knybel et al have also shown that the lead can exhibit larger motion than the target area, and that the correlation between lead motion and target motion vary from patient to patient.^25^ Both cardiac gating under voluntary breath hold and combined cardio‐respiratory gating have been shown to be feasible in preclinical studies,^26^, ^27^ and further investigation of these techniques may offer a way to reduce total cardio‐respiratory positioning uncertainty. All of these techniques, however, come at the cost of increasing treatment time for a patient population whose cardiopulmonary reserve is already prohibitive of a supine position. More sophisticated approaches to time‐correlated CT which rely on physical properties in the lung and heart may lead to more accurate motion modeling.^28^ Finally, MR‐based target tracking has been suggested for treatment in the thorax,^29^ and has been suggested for radioablation for atrial fibrillation.^30^ It remains to be seen how effective target tracking will be in VT patients receiving CR where ICD leads may induce artifacts in cine MR images of the heart.

Respiratory‐correlated 4DCT is prone to artifacts based on irregular breathing patterns, and these artifacts have been shown to affect clinical outcomes.^31^ When treating the heart, where motion is more erratic, the impact of these artifacts is not fully understood. During 4DCT, the pitch of the CT table is reduced and several images at each phase of the breathing cycle are collected. Target definition, normal structure delineation, and dose calculation are typically carried out on the respiratory phase‐averaged CT scan that blurs motion. Because the cardiac cycle is much faster than the respiratory cycle, these averaged 4DCTs encapsulate several cardiac cycles, generating an image below the temporal resolution required to delineate cardiac CTVs. Additionally, because the cycles are asynchronous, there is no guarantee that successive slices in individual phase‐gated CTs will show subregions of the heart in the same position. With ECG‐gated CT this problem is eliminated and target volumes can be accurately delineated on each phase or at maximum systole and maximum diastole. In the context of treatment planning, ECG‐gated CT has been used prospectively to create an ITV which was transferred to the treatment planning CT for planning, reducing concerns about motion blurring.^25^, ^32^ Our method of DVF‐propagated CTV generation to estimate motion in this work partially automates this process, allows for voxel‐specific motion estimates, and could be used to design ITVs with proper DIR quality assurance. While the use of ECG‐CT based ITVs may help to account for cardiac motion, this approach will not work for all CR patients, as many are unable to tolerate intra‐venous contrast due to their clinical status which many times includes compromised kidney function. Additionally, the ITV must be implemented with caution as this will increase treatment volumes, and in patients with larger VT substrates this may lead to unacceptable post‐treatment toxicity.

As with other RT treatment sites,^33^ delineation of CR target volumes is a large source of uncertainty which is compounded by the need to include multiple modalities, including non‐3D techniques such as ECGs.^12^, ^34^ While there are multiple proposed methods for target delineation,^4^, ^10^, ^35^, ^36^, ^37^ until a uniform consensus is reached, the uncertainty from target delineation may impact treatment more than cardiac motion. Our clinical practice in this patient cohort explicitly considers the inherent uncertainty in target delineation arising from electroanatomical transfer mapping, 4DCT temporal resolution, and cardiopulmonary motion. Many of the target volumes were contoured conservatively large in a consensus process incorporating expert input across cardiology, radiation oncology, radiation physics, and dosimetry on a case‐by‐case basis. While the drawn target volume may be underdosed in the treatment planning system, the physical dose to the true VT substrate may receive full dose.

In many motion studies, CMD is the metric used for reporting structure shifts,^13^, ^14^, ^15^, ^38^, ^39^ while in this study we relied on using the DVF. In this patient cohort, mean value of the DVF and CMD were close, however the DVF typically produced larger motion estimates. While the CMD reports only one number, the DVF is an entire field so additional quantitative metrics and tools can be used for further analysis. The DVF also provides qualitatively useful information when motion is multi‐directional across the volume of interest, as for the targets that wrapped around the left ventricle as shown in Figure 2.

The magnitude of cardiac motion in this study was smaller than reported in previous studies. Morris et al used cardiac MRI to study motion in the chambers prior to irradiation in lung cancer patients.^13^ They found median motion magnitudes of 5.2 mm for the ventricles and 5.1 mm for the atria. In this study, using the DIR‐generated contours, median motion measured by mean DVF was 4.5 mm and 3.9 mm for the atria and ventricles and using the manual contours, 4.9 mm and 4.0 mm as measured with CMD. In a study using ECG‐gated CT, Ouyang et al measured motion for the left and right ventricles using CMD.^15^ SI motion was much smaller than that found in this study with 0.51 mm for the LV and 1.00 mm for the RV, compared with 2.72 mm and 1.86 mm (DVF), and 1.20 and 2.29 (CMD). Finally, Prusator et al used rigid registration to assess cardiac motion.^17^ While rigid registration cannot capture the deformation of the heart during the cardiac cycle, the authors minimized the impact of this deformation by focusing the registration on the target volume and surrounding myocardium. The mean (range) cardiac motion found in their cohort of 11 patients was 3.4 (1.0‐4.8) mm ML, 4.3 (2.6‐6.5) mm AP, and 4.1 (1.4‐8.0) mm SI, as compared to 1.7 (0.0‐5.2) mm ML, 2.4 (0.5‐3.9) mm AP, and 2.1 (0.4‐4.6) mm SI. While the magnitude of motion in each study was different, both studies found that cardiac motion can play a significant role in CR and that respiratory 4DCT may not fully encapsulate target motion. This is consistent with the findings of Knybel et al, who employed online tracking during treatment delivery and found that target motion exceeded 3 mm in 35% of their cases.^25^

A central limitation of this study is reliance on DIR. DIR performance assessment is a subjective process which is dependent on user experience and can be subject to error without quality assurance (QA). The registration QA also needs to be specific to the underlying goal of the DIR.^40^ Different DIR algorithms can also produce different results,^18^, ^41^ so the validity of the results of a DIR‐based study rely on accurate benchmarking of the DIR algorithm, as specifically evaluated for a given application. As the goal of DIR in this work was to generate contours that could be used for shift calculations, we set to first verify the contour accuracy and then the accuracy of shift calculations. The comparisons between manual and DIR‐derived cardiac chamber contours shown in the supplementary materials indicate that the overlap is imperfect, with an average DSC of 0.86, but the average difference in contour position is 1.7 mm, and average difference in cardiac chamber shifts is relatively small (0.5 mm) and indicates strong agreement for motion measurement. With post‐processing, it is possible that these results could be further improved. The calculation of dose on these deformed image sets also includes inherent uncertainty due to the spatial registration. This is further exacerbated because only systole and diastole images were used, and the details of cardiac cycle timing and respiratory motion were neglected. Rather than representing the dose these patients received, these calculations show that tight target margins typical of SBRT can lead to dose deviations even in the presence of relatively small motion.

All dose calculations in this work were performed with AAA, which has known limitations, especially near heterogeneities such as the cardiopulmonary interface.^42^, ^43^ As more accurate grid‐based Boltzmann solver (GBBS) dose calculation algorithms, such as Acuros XB (Varian Medical Systems), become more widespread, there may be limited applicability of these results to other studies where GBBS algorithms are used. However, AAA is still the standard dose calculation on our clinic, and the trend of decreased dose coverage due to larger or irregular motion is not likely to decrease when a different dose calculation algorithm is used.

This single‐institution retrospective analysis included only eight patients. With low patient numbers, and given the variable motion patterns found, it is difficult to generalize these results without further research. However, given the limited number of patients who have been treated worldwide with this technique, we believe this is an adequate sample size to conclude that cardiac motion should be considered during CR treatment planning. This study, along with that by Prusator et al, show anatomic location is important in terms of both magnitude and direction of motion. We believe that the data shown in this work underscore the need for greater investigation into the complex interplay of cardiorespiratory motion and target geometry to inform robust motion protocols moving forward. In future trials, ECG‐gated CT may be integral for treatment planning so that target motion can be fully encapsulated for each patient to ensure the full target volume is treated.

## 5 CONCLUSIONS

Cardiac motion as measured by ECG‐gated CT in a cohort of patients treated with CR was analyzed and using a DIR‐based methodology, the dosimetric impact of this motion was studied. For five out of eight patients, the motion was small and target coverage was not greatly impacted; however, for the remaining three patients, cardiac motion may have decreased the percentage of the contoured target volume receiving prescription dose. Both the magnitude and direction of motion were dependent on target location. To the best of our knowledge, this is the first study of cardiac target motion and its potential dosimetric implications.

## AUTHOR CONTRIBUTIONS

Drs. Lloyd, Higgins, and Castillo contributed to the conception of this work and designed the experiments. Drs. Harms, Schreibmann, and McCall put forth significant effort in acquisition and analysis of the data. All authors contributed the interpretation of the data, as well as the writing, preparation, and editing of the manuscript. All authors have given final approval of this submission and agree to be accountable for all aspects of the work in ensuring that questions related to the accuracy or integrity of any part of the work are appropriately investigated and resolved.

## CONFLICT OF INTEREST STATEMENT

The authors have no conflicts of interest relevant to this work to disclose.
